# The Use of Disposable Tampons as Visual Biofeedback in Pelvic Floor Muscle Training

**DOI:** 10.3390/ijerph16122143

**Published:** 2019-06-17

**Authors:** María Zahara Pintos-Díaz, Paula Parás-Bravo, Cristina Alonso-Blanco, César Fernández-de-las-Peñas, María Paz-Zulueta, Mónica Cueli-Arce, Domingo Palacios-Ceña

**Affiliations:** 1 Pelvic Floor Unit, Department of Rehabilitation, Hospital 12 Octubre, Madrid Health Service, Avda. Cordoba s/n, 28041 Madrid, Spain; zahara.pintos@salud.madrid.org; 2 Faculty of Nursing, University of Cantabria, Avda Valdecilla s/n, 39008 Santander, Cantabria, Spain; maria.paz@unican.es (M.P.-Z.); monica.cueliarce@unican.es (M.C.-A.); 3 Nursing Research Group, Health Research Institute IDIVAL, c/Cardenal Herrera Oria s/n, 39011 Santander, Cantabria, Spain; 4 Department of Physiotherapy, Occupational Therapy, Rehabilitation, and Physical Medicine, University Rey Juan Carlos, Avda de Atenas s/n, 28922 Alcorcón, Madrid, Spain; cristina.alonso@urjc.es (C.A.-B.); cesar.fernandez@urjc.es (C.F.-d.-l.-P.); domingo.palacios@urjc.es (D.P.-C.); 5 Health Law and Bioethics Group, Health Research Institute IDIVAL, c/Cardenal Herrera Oria s/n, 39011 Santander, Cantabria, Spain

**Keywords:** Tampon, muscle strength, pelvic floor, biofeedback, urinary incontinence

## Abstract

*Background*: Urinary incontinence represents a complex problem which commonly affects women and influences their physical, mental and social wellbeing. The objective was to determine the effect of pelvic floor muscle training using a tampon as visual biofeedback. *Methods*: A non-randomized clinical trial involving 60 women >18 years of age, both with, and without, urinary incontinence. All women exercised with a program involving visual biofeedback using disposable tampons at home for three months. The compliance rate was 76.8 ± 24.1 An electromyographic assessment of the pelvic floor was performed and assessments of the impact of the exercise program. *Results*: 54.5% of women without incontinence and 81.6% of women incontinence reported improvements (*p* = 0.041). In both groups, there was increased quality life (*p* > 0.05). The women without incontinence experienced greater improvement in the quality of their sexual relations (Pre 6.8 ± 1.4–Post 7.2 ± 1.0). *Conclusions*: After the intervention, a high percentage of women showed a statistically significant improvement in their symptoms. The participants reported an increase in quality of life and the women without incontinence reported an improvement in quality of their sexual relations. Our findings suggest that visual BFB for training the PFM may be beneficial for women with or without incontinence.

## 1. Introduction

Female pelvic floor dysfunction (FPFD) is defined as alterations caused, either totally or partially, by poor functioning of pelvic structures or by structural alterations of the pelvic floor muscles (PFM), ligaments or nerves [1]. Among these dysfunctions, urinary incontinence (UI), which is defined by the International Continence Society (ICS) as the complaint of any involuntary leakage of urine [2,3], represents a common problem for women.

The prevalence of UI varies considerably depending on several factors, including age, the number of births, and the presence of comorbidities. It has been estimated to be around 15–17% in mid-age women [4]. Nevertheless, it may be lower (10.3%) in younger women and teenagers [5], and higher in women older than 70 years old [6] or obese women [7], reaching 36.2% or 48.4%, respectively. UI is associated with a decrease in physical, mental and social well-being caused by a decrease in the overall and sexual quality of life, by a decrease in self-esteem, and by the presence of anxiety and embarrassment due to the odor and the unpredictability [8].

It seems that stress urinary incontinence (SUI), the complaint of an involuntary leakage of urine during effort or physical exertion [2], and mixed urinary incontinence (MUI), the complaint of involuntary leakage of urine associated with urgency and also with effort during physical exertion or with sneezing or coughing [9,10], are the most common types of UI. Urgency urinary incontinence (UUI), the complaint of involuntary leakage of urine associated with urgency, is the third most prevalent [2].

Among different treatment options for the management of PF dysfunctions, proper training of the Pelvic Floor Muscles (PFM) seems to be the most effective intervention, particularly for SUI [11], although further studies are clearly needed [12]. Exercises for PFM tend to be more effective when combined with biofeedback (BFB) [13,14]. In fact, the use of BFB is highly common in rehabilitation programs for fibromyalgia syndrome, cerebrovascular disease, asthma, hypertension, or heart failure [15]. Original studies by Kegel found an improvement in 84% of women with UI using a protocol that included vaginal palpation and observation of voluntary PFM contractions with BFB of vaginal pressure during the exercises [16]. Posteriorly, Kegel developed the perineometer [17]. Burns et al. [18] observed that the use of BFB helps for increasing the coordination and control of the PFM compared to the performance of exercises alone.

Nevertheless, the use of BFB equipment can represent an elevated cost for the patient; therefore, more economical instruments should be used for providing feedback. An accessible, extended and cheap instrument that could be easily used for providing feedback during PFM exercises is the tampon. No previous study has investigated whether this instrument can be used for providing feedback during PFM exercises. In addition, most studies only investigate the effects of exercises in women with urinary incontinence, so the possible effects or benefits on women without incontinence are unknown. Therefore, the aim of the current study was to investigate the effects of PFM training using a tampon as visual biofeedback on muscle competence, self-reported quality of life, and quality of sexual relations in women with and without UI. We hypothesized that PFM exercises using a tampon as a feedback will be more effective for improving muscle competence and other variables in women with UI than in those without UI.

## 2. Materials and Methods

### 2.1. Study Design

A non-randomized clinical trial was conducted where women were divided based on the presence or absence of UI. All women received the same PFM exercise program by using a tampon as a visual feedback. The study design was approved by the Ethics Committee of the University Rey Juan Carlos, Spain. All procedures were conducted according to the Declaration of Helsinki and participants read and signed a written consent form prior to their participation in the study.

### 2.2. Participants

The study was carried out in a physiotherapy clinic specializing in gynecological care (Madrid, Spain). Women older than 18 years from January 2014 to January 2016 were invited to participate. They were grouped by the presence or absence of UI. They were excluded if they met any of the following criteria: 1, pregnancy; 2, post-natal situation (up to 3 months after delivery); 3, pelvic organ prolapse; 4, persistent urinary tract infections; 5, any systemic condition, e.g., fibromyalgia syndrome, arthritis rheumatoid; 6, mental disorder; 7, neurological condition; or, 8, had received physical therapy intervention within the pelvic area in the previous year. Socio-demographic and gynecological variables were collected as follows: age, profession, body mass index (BMI), type of UI, number of births, and episiotomy.

### 2.3. Electromyographic (EMG) Assessment

**Figure 1:**
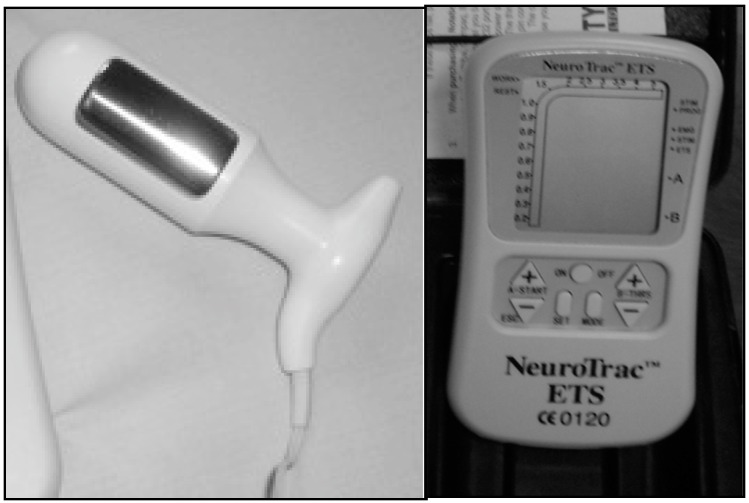
The veriprobe vaginal electrode and the NeuroTrac ETS stimulation device (source: author’s photograph).

**Figure 2:**
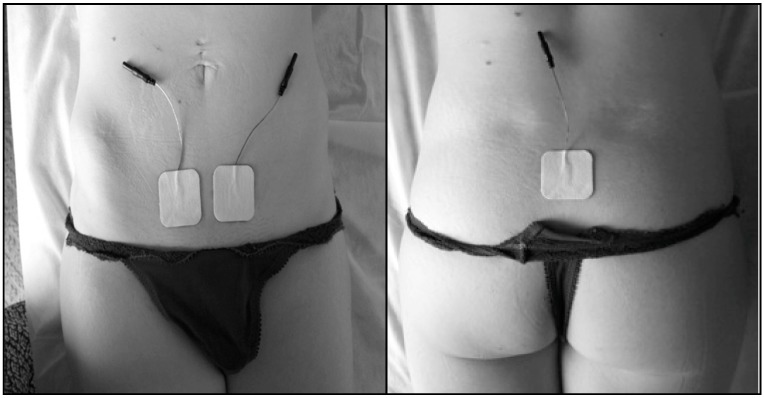
Electrode placement at the abdominal and sacral levels (source: author’s photograph).

EMG assessments were conducted using the NeuroTrac^TM^ ETS equipment (Verity Medical Ltd, Romsey, UK) (Figure 1) with the following electrode placement: 1, intra-vaginal [19,20] Veriprobe was inserted in the vagina for assessing the electromyographic activity of the PFM; 2, two superficial electrodes (size: 40 × 40 mm) were placed symmetrically at a half distance from both sides of the mid-abdominal line and one centimeter above the pubic bone to monitor the rectus abdominis muscle; and, 3, a reference electrode (size: 40 × 40 cm) was placed at the level of the sacrum (Figure 2).

The electromyographic assessment was performed both in supine position with triple lower limb flexion (ankle, knee and hip flexion), as well as standing. In both assessments, the participants were asked to perform five series of ten repetitions of phasic contractions and five repetitions of tonic contractions lasting ten seconds with ten seconds rest in between. Regarding the phasic contractions, we evaluated their average value, the number of valid contractions and the ratio between the average value of the phasic contractions and the average value of the abdominal activation that these provoked. Regarding tonic contractions, we assessed the average value, the base and the ratio between the average value of the tonic contractions and the average value of the abdominal activation that these provoked.

EMG outcomes were assessed with participants placed in supine and in standing positions. PFM activity was evaluated as follows: 1, mean value of phasic contractions and valid phasic contractions (these must be less than 1 s’s width and peak of the needle); 2, relation between the mean value of phasic contractions and the mean value of the abdominal contraction conducted by the participant in response to the phasic contractions of the PFM (phasic synergy); 3, the mean value of tonic contractions; 4, tonic base (duration in seconds of the tonic contraction); and, 5, the relation between the mean value of the PFM tonic contraction and mean abdominal value that the participant performs in response to the tonic contractions of the PFM (tonic synergy).

### 2.4. Quality of Life, Sexual Relations and Motivation

Although there are various questionnaires evaluating the health-related the quality of life [21,22], they are highly specific and did not assess the general impact of FPFD on the quality of life of these women. Furthermore, in this study, woman with and without UI were included. Therefore, we decided to collect data related self-perceived quality of life, quality of sexual relationships and the degree of motivation for participation in the current study with a numerical rate scale ranging from 0 to 10 points.

### 2.5. Intervention

An ad hoc exercise program was designed to be carried out at home. The supine decubitus with the lower extremities in flexion. A tampon was introduced in the vagina with the applicator extended. The exercise program had two parts: (1) 5 series of 10 phasic contractions with a 10 sec rest-period between series. (2) 5 tonic contractions lasting 10 s with a 10 s rest-period in between. Participants were able to see the movement of the buffer in a mirror during contraction, providing them with visual feedback, which facilitated the ability to self-correct the exercise (visual BFB). A proper contraction of the PFM was considered when the outer tip of the buffer moved in a downwards direction during the contraction. An inappropriate contraction of the PFM was considered when the buffer did not move, moved upwards or came out during contraction. The frequency of the exercise protocol was once a day.

An appropriate number of disposable tampons was provided to each participant to perform the exercise program over a three-month period.

### 2.6. Procedure

Data collection was performed at baseline and after the treatment period (three months) by an assessor blinded to the assigned group (UI or non-UI).

After baseline data collection, an inspection of the PFM consisting of a vaginal manual examination assessing sensibility, presence of prolapsed, muscle tone and strength (EMG) was performed by a physical therapist with more than 10 years of experience in UI treatment.

Lastly, the patients were trained in the performance of PF exercises in supine position with triple flexion of the lower limbs using a tampon with a conventional applicator as a visual BFB device (see Section 2.5. Intervention).

### 2.7. Statistical Analysis

The data was analyzed with the SPSS version 18.0. statistical package (SPSS Inc., Chicago, IL, USA). For the descriptive analysis, the mean and standard deviations were calculated. The Kolmogorov-Smirnov test was performed for the assessment of the normal distribution of the quantitative variables (*p* > 0.05). The results displayed a normal distribution of all the quantitative variables and, therefore, parametric tests were performed for the statistical analysis. The Student’s t-test for independent samples was used in order to compare the values of the quantitative variables of each group, prior to the intervention, in order to determine whether these were comparable.

To analyze the changes obtained in each of the groups, a two-way analysis of variance (ANOVA) was performed on the quantitative variables analyzed. The group factor was introduced (with or without UI) as an inter-subject factor and the time factor (before or after the intervention) was used as an intra-subject factor. The main analysis was centered on the group interaction by time for each variable.

For the qualitative variables, the difference in the distribution of responses before and after treatment was analyzed with the extended Chi squared test (χ^2^).

The statistical analysis was performed with a confidence interval of 95%; therefore, statistically significant values were considered to be those with *p* < 0.05.

## 3. Results

Demographic and clinical data of the patients: Table 1

A total of 60 women participated; these were divided into two groups according to whether or not they had UI in Table 1.

In total, 60 women aged between 20 and 65 years participated in the study, with a mean age of 47.3 ± 11 years. 36.37% of the women (*n* = 22) declared that they did not suffer UI. Up to 63.3% of the sample (*n* = 38) declared having experienced some type of urinary symptom: 55.3% had symptoms of SUI, 15.8% presented UUI, 10.5% had MUI, 5.3% had chronic urinary retention, and 5.3% had postpartum UI, whereas 7.9% were unable to determine which type of UI they suffered from. By age, UI was suffered by 5.3% of women aged between 20 and 34 years old, 47.4% of those aged between 35 and 49 years, and 47.4% of women between 50 and 65 years old.

The mean value of the BMI was 25 ± 4.7, which corresponds to a normal weight, albeit very close to being overweight. Women without UI had a BMI of 24.2 ± 3.9 and those with UI had a mean BMI of 25.5 ± 5.1.

Of the total of women without UI, 68.2% had given birth, compared to 89.5% of those who were incontinent (*p* = 0.040), with the mean global number of births equaling 1.6± 1.1 births.

After the performance of the proposed exercise protocol, 54.5% of women without UI and 81.8% of women with UI declared having improved (*p* = 0.041). The average compliance was 76.8 ± 24.1 (*p* < 0.001).

Up to 52.6% of women with UI symptoms had performed some kind of previous exercise prior to the intervention; these reports included PF contractions, yoga, hypoppresive exercises and pipistop (detection of voluntary urination in order to reveal the contraction of the PFM and use it as a kind of BFB). Six of the women without UI and 12 of those with UI who reported having performed exercises were unable to determine which type of exercise they had performed.

Up to 70% of these considered that the PFM training protocol with the tampon was better than any previous methods they had performed. For 95.5% of women without UI and 79% of those who were incontinent, the duration of the proposed protocol was appropriate. The study met the expectations of 54.5% of the women without UI and 68.4% of those who were incontinent.

Electomyogram, quality of life and sexual relations: Table 2.

Table 2 displays the results of the ANOVA comparing the initial assessment with the final assessment.

The ANOVA did not reveal any group by time interactions for any of the variables in supine position: phasic mean (F = 0.020; *p* = 0.888), valid phasic contractions (F = 0.004; *p* = 0.950), phasic synergies (F = 0.942; *p* = 0.336), tonic mean (F = 0.008; *p* = 0.931), tonic stability (F = 0.030; *p* = 0.863), tonic base in supine (F = 0.646; *p* = 0.425), or tonic synergy (F = 0.149; *p* = 0.701). In this way, both groups obtained similar changes in all the variables after the tampon exercise program. Similar results were found in the same variables in standing position: phasic average (F = 1.398; *p* = 0.243), phasic valid contractions (F = 0.029; *p* = 0.864), phasic synergies (F = 2.063; *p* = 0.157), tonic mean (F = 1.771; *p* = 0.189), tonic base in supine (F = 1.340; *p* = 0.252), or tonic synergy (F = 1.813; *p* = 0.184). Once again, the changes obtained were similar in both groups.

Regarding variables such as the impact on quality of life (F = 0.564; *p* = 0.456), quality of sexual relations (F = 0.836; *p* = 0.365), and motivation (F = 0.003; *p* = 0.957), once again the ANOVA failed to find a significant interaction between groups by time. Nevertheless, the women without UI experienced a greater improvement in the quality of their sexual relations (women without UI Pre 6.8 ± 1.4—Post 7.2 ± 1.0 vs. women with UI Pre 6.3 ± 2.5—Post 6.6 ± 2.3) compared to those who suffered UI.

## 4. Discussion

Although the ICS establishes a unified terminology for describing FPFD [2], there is a great heterogeneity in the scientific literature due to the growing complexity of FPFD [2,23].

In our study, 63.33% of women who participated declared that they had suffered some type of UI. This finding is much higher than previous reports. This may be due to the fact that the women participating in the study were recruited from a physiotherapy clinic specializing in gynecological care, and therefore the data are state inflated and may not be comparable to the general public. In a recent literature review, Dieter et al. [4] concluded that between 15 and 17% of women suffered from UI. Zekele et al. [6] estimated a prevalence of 36.2% in a sample of women whose mean age was over 70 years. At the opposite extreme, Parden et al. [5] reported a prevalence of 10.3% in teenagers and young women. Al-Mukhtar Othman et al. [7] reported that the prevalence of UI was between 9.7 and 48.4% according to the BMI of the women studied. These differences regarding epidemiological data are due, in part, to the fact that the UI varies according to the women’s age and the presence of other comorbidities such as obesity, which is intimately related with FPFD, among others [23,24].

Of the women with UI, 55.3% had SUI, 15.8% had UUI, 10.5% had MUI, 5.3% had chronic urinary retention and 5.3% had postpartum UI, whereas 7.9% did not know how to determine what type of UI they suffered from. Ghafouri et al. [10] found that 41.9% had symptoms of SUI, 44.5% had MUI, 13.6% had UUI. Meanwhile, Akkus et al. [9] reported that 37.7% had symptoms of SUI, 59.2% had MUI, 3.1% had UUI. Once more, we find disparity in the data due to the difficulties surrounding the adoption of a unified criteria and the previously commented associated factors [2,23].

In this study, 68.2% of the women without UI had given birth, compared to 89.5% of incontinent participants (*p* = 0.04), which supports the relation between giving birth as a risk factor for suffering UI [25].

Up to 50% of women without UI and 81.57% of those with UI reported improvements (*p* = 0.018) although improvements were not detected in the electromyographic variables, despite the high compliance of the exercise protocol on behalf of the participants.

Other authors have reported statistically significant differences in women performing PFM training, achieving a greater contractibility of the muscles as well as a decrease of the urinary symptoms [19]. In this sense, another study revealed that high intensity pelvic floor training together with training of the bladder is more effective than bladder training alone [26].

Celiker [27] achieved a reduction in UI symptoms with a program of PFM training up to a score of 5 in the Oxford scale, obtaining a statistically significant reduction of UI and an increase in muscle strength.

Although the patients experienced a qualitative increase to their perceived quality of life, this increase was not statistically significant. Notwithstanding, the high motivation for participating in the study brings to light the profound impact that FPFD has on the participants’ overall quality of life, sexual quality of life, and self-esteem, among other things, as reported by other authors [8].

Regarding sexual activity, women without UI experienced a higher quality of their sexual relations, although this improvement was not statistically significant. These results are supported by previous studies, which failed to achieve statistically significant improvements in the quality of sexual relations via PFM training programs [28].

A recent Cochrane review [12] revealed that there is insufficient evidence for recommending the addition of PFM training to other treatments for UI in women (physical therapy, behavioral therapy, drugs, or even surgery, among others). However, the previously commented studies with specific training protocols seem to be effective at improving UI symptoms.

In our study, the hypothesis that PFM training using a disposable tampon as a visual BFB improves the competency of the PFM warrants further research with larger samples.

### Limits of the Study

This research was centered specifically on UI as this is the most prevalent FPFD. It would therefore be convenient to study the effect of the tampon as visual BFB and for training of PFM in each of the related dysfunctions. The use of the tampon by participants as a type of visual BFB may produce a variability in the results due to the use, valuation of the same and subjective factors related to its use (past experience, beliefs, etc.). On the other hand, the width of the subcutaneous tissue, the skin resistance, the vaginal impedance (which may vary due to the menstrual cycle) and the position of the electrodes [20] are factors that may have caused a certain variability in the results when performing a comparison using BFB.

Furthermore, we did not use a validated questionnaire for quality of life, due to the absence of an appropriate questionnaire suited to the characteristics of this study.

Certain factors that depend on the participants themselves may have influenced the effect of PFM training on FPFD, such as the degree of incontinence, age, the integrity of sphincters, the perineal tonus, the surgical history and radiological findings, patient motivation, consistency [23] and the integrity of pelvic floor (cicatrix surgical or episiotomy for example).

The study did not have a control group.

Finally, another limitation of this study was the small number of participants. Therefore, to confirm our findings future studies with a bigger sample and a control group are necessary.

## 5. Conclusions

After the intervention, a high percentage of women (without and with UI) showed a statistically significant improvement in their symptoms. The participants of both groups reported an increase in the quality of life that was not statistically significant. Women without UI reported an improvement in quality of their sexual relations that was not statistically significant. Our findings suggest that visual BFB for training the PFM may be beneficial for women with or without UI. However, to confirm our findings it is necessary to conduct studies with a bigger sample in the future.

## Figures and Tables

**Table 1:** Demographic and gynecological data.

	*n* = 60	Mean (SD)	Women without UI		Women with UI		*p* *
			*n* = 22	%	*n* = 38	%	
Age, years	20–34	47.3 (11)	7	31.8%	2	5.3%	0.004
	35–49		3	13.6%	18	47.4%	
	50–65		12	54.5%	18	47.4%	
Incontinence symptoms	Stress		.	.	21	55.4%	
	Urgency		.	.	6	15.8%	
	Mixed		.	.	4	10.5%	
	Chronic urinary retention		.	.	2	5.3%	
	Postpartum		.	.	2	5.3%	
	DK/NR		.	.	3	7.9%	
BMI, kg/m^2^	Underweight	25 (4.7)	1	4.5%	-	-	0.446
	Normal weight		13	59.1%	20	52.3%	
	Overweight		6	27.3%	15	39.5%	
	Obesity		1	4.5%	3	7.9%	
Educational level	Low		13	59.1%	19	50%	0.79
	Medium		6	27.1%	13	34.2%	
	High		3	13.6%	6	15.8%	
Have you given birth?	No		7	31.8%	4	10.5%	0.04
	Yes		15	68.2%	34	89.5%	
Number of births	0	1.6 (1.1)	7	31.8%	4	10.5%	0.071
	1		4	18.2%	11	29%	
	2		7	31.8%	19	50%	
	3		2	9.1%	4	10.5%	
	4 or more		2	9.1%	-	-	
Episiotomy	No		-	-	4	11.8%	0.166
	Yes		15	100%	30	88.2%	
Do you think you have improved	No		9	42.9%	7	18.4%	0.041
	Yes		12	54.5%	31	81.6%	
	DK		1	2.2%	-	-	
Previously, did you exercise?	No		12	54.5%	18	47.4%	0.592
	Yes		10	45.5%	20	52.6%	
Type of previous exercise	Pipistop		2	20%	4	20%	0.622
	PF contractions		1	10%	3	15%	
	Yoga		1	10%	-	-	
	Hypopressive exercise		-	-	1	5%	
	DK		6	60%	12	60%	
Comparison with other previous methods	Better		7	70%	15	75%	
	The same		2	20%	-	-	
	Worse		-	-	1	5%	
	DK		1	10%	4	20%	
Classification of the treatment time in the third session?	Too little		-	-	7	18.4%	0.107
	Appropriate		21	95.5%	30	79%	
	A lot		1	4.5%	1	2.6%	
Have your expectations been met?	No		8	36.4%	5	13.2%	0.064
	Yes		12	54.5%	26	68.4%	
	In part		-	-	5	13.2%	
	DK		2	9.1%	2	5.3%	

* Chi-Squared Test; UI: Urinary incontinence; SD: Standard deviation; PF: pelvic floor; DK: Do not know.

**Table 2:** Comparison between the initial and final assessment via the ANOVA test.

Position	Mensuration	Women without UI *n* = 22		Women with UI *n* = 38		
		Pre	Post	Pre	Post	*p* *
Supine position	Phasic mean ^b^	52.5 ± 34.6	50.3 ± 37.1	62.6 ± 38.5	59.6 ± 37.6	0.888
	Valid phasic contractions ^c^	59.8 ± 27.3	75.6 ± 16.7	52.1 ± 25.7	68.4 ± 22.6	0.95
	Phasic synergy ^d^	5.1 ± 3.1	5.9 ± 3.6	6.3 ± 4.5	7.5 ± 5.6	0.336
	Tonic mean ^e^	29.3 ± 17.3	35.2 ± 19.3	40.8 ± 24.8	47.1 ± 36.7	0.931
	Tonic stability ^f^	62.4 ± 11.6	66.6 ± 9.7	59.5 ± 14.1	64.3 ± 11.9	0.863
	Tonic base ^g^	7.9 ± 1.9	7.4 ± 1.4	8.3 ± 1.8	7.3 ± 1.8	0.425
	Tonic synergy ^h^	3.6 ± 2.3	4.6 ± 3.3	5.6 ± 4.1	6.1 ± 3.8	0.701
Standing	Phasic mean ^b^	51.7 ± 32.3	48.9 ± 28.2	58.5 ± 32.1	47.7 ± 25.1	0.243
	Valid phasic contractions ^c^	52.7 ± 22.8	66.1 ± 23.2	48.5 ± 26.3	63.0 ± 26.6	0.864
	Phasic synergy ^d^	3.9 ± 2.6	4.5 ± 2.7	4.3 ± 2.9	3.7 ± 2.5	0.157
	Tonic mean ^e^	35.7 ± 20.7	37.8 ± 20.7	40.8 ± 24.3	35.7 ± 20.7	0.189
	Tonic base ^g^	7.3 ± 1.7	7.2 ± 1.4	7.9 ± 1.3	7.4 ± 1.7	0.252
	Tonic synergy ^h^	3.2 ± 1.8	3.5 ± 2.3	4.6 ± 4.9	3.4 ± 2.3	0.184
Impact on quality of life ^a^		5.6 ± 2.9	6.4 ± 2.6	6.1 ± 2.1	6.4 ± 2.3	0.456
Impact on quality of sexual relations ^a^		6.8 ± 1.4	7.2 ± 1.0	6.3 ± 2.5	6.6 ± 2.3	0.365
Motivation for participating in the study ^a^		8.4 ± 1.6	7.9 ± 2.1	8.8 ± 1.3	8.3 ± 2.0	0.957

* Chi-Squared Test. PF Pelvic Floor; ^a^ Scale from 0 to 10; ^b^ Five series of ten phasic contractions, with ten seconds rest between series and without rest between phasic contractions. Each of these were assessed and afterwards the mean value was calculated; ^c^ A valid contraction has a base of less than one second width and needle peak; ^d^ Relation between the mean value of phasic contractions and the mean value of the abdominal contraction that the patient performs in response to the phasic contractions of the PF; ^e^ Five tonic contractions with a base of 10 s with 10 s rest between each of the tonic contractions; ^f^ Stability of the strength of the tonic contraction force; ^g^ Number of mean seconds duration of tonic contractions; ^h^ Relation between the mean value of tonic PF contractions and the mean abdominal value.
